# Exploring Ferroptosis-Associated Gene Signatures as Diagnostic and Therapeutic Targets for Sepsis-Induced Cardiomyopathy

**DOI:** 10.7759/cureus.60439

**Published:** 2024-05-16

**Authors:** Haobin Huang, Chenbo Ge, Yawei Dai, Yanhu Wu, Jinfu Zhu

**Affiliations:** 1 Cardiovascular Surgery, The First Affiliated Hospital of Nanjing Medical University, Nanjing, CHN

**Keywords:** sepsis-induced cardiomyopathy, cerna network, therapeutic targets, bioinformatics, ferroptosis

## Abstract

Background: Sepsis-induced cardiomyopathy (SICM) is a severe complication of sepsis associated with high mortality rates. Despite its significance, the molecular mechanisms underlying SICM remain poorly understood, particularly the role of ferroptosis - a form of iron-dependent programmed cell death.

Methodology: This study analyzed the GSE79962 dataset from the Gene Expression Omnibus, containing cardiac gene expression profiles from SICM patients and controls. A list of ferroptosis-related genes (FRGs) was retrieved from the FerrDb. We used the *limma* package in R for differential expression analysis, setting an adjusted *P*-value cutoff of <0.05 and a log2-fold change threshold of ±1 to identify differentially expressed ferroptosis-related genes (DE-FRGs). We applied machine learning algorithms for biomarker identification, including least absolute shrinkage and selection operator (LASSO) logistic regression and support vector machine with recursive feature elimination (SVM-RFE), implemented via the *glmnet* and *e1071* packages in R, respectively. Gene set enrichment analysis (GSEA) was conducted using the *GSEA* package to investigate the biological pathways related to key DE-FRGs.

Results: After differential expression analysis, we identified 145 DE-FRGs. Functional enrichment analyses underscored the involvement of these genes in critical biological processes and pathways, such as lipid metabolism and insulin resistance. Machine learning approaches pinpointed five key DE-FRGs (NCOA4, GABARAPL1, GJA1, CISD1, CP), with strong predictive potential for SICM. Further analyses, including the construction of a ceRNA network, revealed intricate post-transcriptional regulatory mechanisms that may influence the expression of these key genes.

Conclusions: Our findings highlight the central role of ferroptosis in SICM and identify potential biomarkers and therapeutic targets that could help refine diagnostic and treatment strategies. This study advances our understanding of the molecular underpinnings of SICM and sets the stage for future research aimed at mitigating this severe sepsis complication.

## Introduction

Sepsis-induced cardiomyopathy (SICM) represents a severe complication of sepsis, significantly escalating mortality rates [1,2]. It is estimated that approximately 40% of septic patients develop myocardial dysfunction [3], a condition that, when present, is linked to a staggering 50% mortality rate [4]. Despite its prevalence and clinical significance, the pathogenesis of SICM remains poorly understood, and there is a pressing need for the development of robust molecular diagnostic techniques and targeted therapeutic approaches to enhance patient outcomes.

Recent insights into iron-mediated programmed cell death, or ferroptosis, have highlighted its crucial role in various pathophysiological conditions [5], including cardiomyopathies linked to sepsis [6]. Ferroptosis is characterized by iron accumulation and lipid peroxidation, leading to cell membrane rupture, thus distinguishing it from traditional cell death mechanisms [5]. Recent studies have elucidated the complex interplay between iron dysregulation and oxidative stress in precipitating ferroptosis within cardiac tissues, significantly contributing to the intricate pathology of SICM [6]. Targeted modulation of cellular processes, particularly through agents like microRNA-130b-3p and the PARP inhibitor Olaparib, has shown potential in mitigating ferroptosis and improving clinical outcomes in SICM models [7,8]. These discoveries underscore the critical role of ferroptosis in SICM pathophysiology and advocate for the exploration of this regulated cell death pathway as a dual-purpose marker for both diagnosis and therapeutic intervention.

The identification of ferroptosis-related genes (FRGs) opens promising avenues for the development of novel diagnostic markers and targeted treatments aimed at mitigating the adverse impacts of SICM. A recent study by Gong et al. utilized animal-derived sequencing data to explore the role of ferroptosis in myocardial injury during sepsis [9]. However, the exclusive reliance on animal models may not fully reflect the human disease context, underscoring the need for studies based on human data. Consequently, this study aimed to validate and extend previous findings through a comprehensive bioinformatics analysis of human data, focusing on the regulatory networks of ferroptosis associated with SICM and their effects on disease progression and treatment responses. Through these efforts, we aspire to enhance the development of effective biomarkers and treatment strategies, enabling early diagnosis and personalized treatment of SICM.

## Materials and methods

Data acquisition

For our analysis, the gene expression dataset GSE79962 was retrieved from the Gene Expression Omnibus (GEO) database hosted by the National Center for Biotechnology Information (NCBI) [10]. This dataset is unique as it contains heart tissue samples from patients diagnosed with SICM, making it the only dataset in the GEO specifically targeting cardiac gene expression in SICM patients. It utilizes the GPL6244 (Affymetrix Human Gene 1.0 ST Array) platform, comparing 20 heart samples from SICM patients with 11 samples from non-failing donor hearts.

To identify FRGs, we utilized the FerrDb [11], which is the world's first database specifically focused on ferroptosis regulators. A total of 728 FRGs were identified for further analysis.

Analysis of differentially expressed FRGs

Expression levels for the 728 identified FRGs were extracted from the GSE79962 dataset. Following data normalization, differential expression analysis was executed using the *limma* package within R software (version 4.3.0). To mitigate the occurrence of false positives, *P*-values were adjusted via the Benjamini-Hochberg procedure. The criteria for identifying differentially expressed ferroptosis-related genes (DE-FRGs) were set to an adjusted *P*-value of <0.05, coupled with a log2(fold change) threshold, where a value greater than 1 indicates upregulation and a value less than 1 indicates downregulation of DE-FRGs in the SICM group. The DE-FRGs were visualized through the construction of a heatmap, facilitated by the ComplexHeatmap package in R.

Functional enrichment analysis of DE-FRGs in SICM

Functional enrichment of DE-FRGs was conducted using the ClusterProfiler package in R. Gene Ontology (GO) and Kyoto Encyclopedia of Genes and Genomes (KEGG) pathway analyses were performed, with significance thresholds set at a *P*-value <0.05, a false discovery rate (FDR) <0.25, and an absolute normalized enrichment score (|NES|) >1.

Machine learning-based identification of key DE-FRGs related to SICM

To identify DE-FRGs associated with SICM diagnosis, we utilized the glmnet package for LASSO logistic regression and the e1071 package for support vector machine with recursive feature elimination(SVM-RFE), incorporating 10-fold cross-validation. A Venn diagram, generated using the *VennDiagram* package in R, was employed for the intersection and visualization of the overlapping genes identified by both approaches.

Single-gene set enrichment analysis (ssGSEA)

Pathways associated with key DE-FRGs were explored using ssGSEA by employing the R GSEA package. The KEGG signaling pathway gene set served as the reference for ssGSEA.

Prediction of potential therapeutic drugs

Potential therapeutic drugs targeting SICM were predicted using the Drug-Gene Interaction Database (DGIdb), which facilitated the identification of interactions between key DE-FRGs and existing pharmacological agents.

Construction of the ceRNA network

To construct a comprehensive ceRNA network, we integrated multiple databases. Initial microRNAs (miRNAs) interacted with key DE-FRGs and were identified using starBase. mRNA sequences for these key DE-FRGs were sourced from NCBI, while human miRNA sequences were downloaded from miRBase. TargetScan, miRDB, and miRanda databases were then employed to predict miRNA target genes, with starBase also used to explore miRNA - long non-coding RNAs (lncRNAs) interactions. This integrative approach enabled the establishment of an elaborate mRNA-miRNA-lncRNA network elucidating the regulatory mechanisms governing ferroptosis within the context of SICM.

## Results

Identification of DE-FRGs

**Figure 1:**
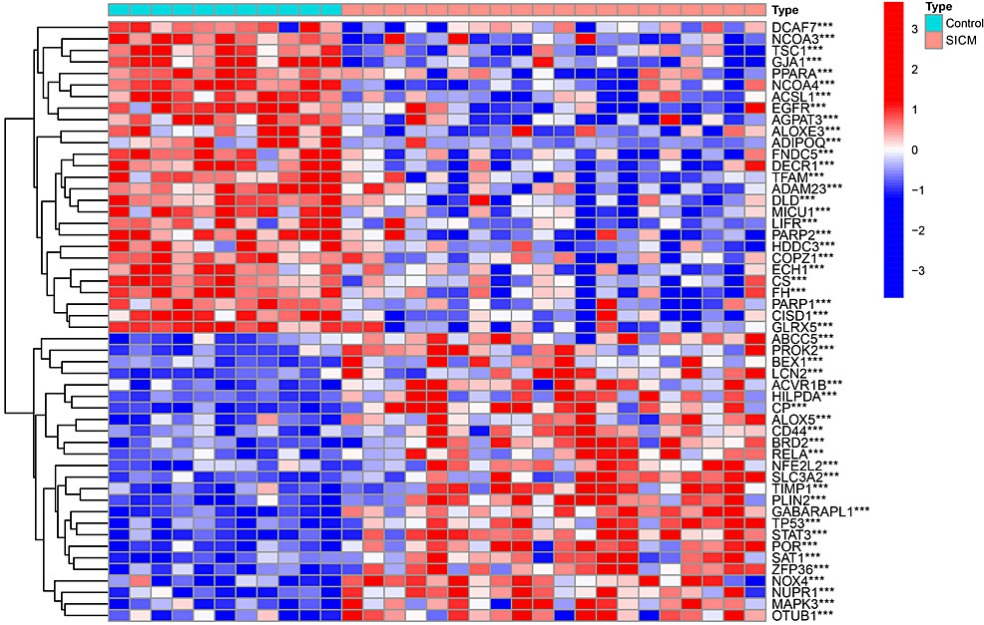
Heatmap of differentially expressed ferroptosis-related genes (DE-FRGs) in sepsis-induced cardiomyopathy (SICM). This heatmap visualizes the expression levels of ferroptosis-related genes that are significantly differentially expressed (*P* < 0.001) between the SICM group and the control group.

We commenced our analysis by identifying 728 FRGs from FerrDb. Subsequent analysis of these genes within the GSE79962 dataset revealed that 145 FRGs exhibited differential expression between the SICM and control groups. Notably, 74 FRGs were upregulated and 71 downregulated in the SICM group, with those exhibiting *P*-values <0.001 depicted in Figure 1.

Functional roles of DE-FRGs

**Figure 2:**
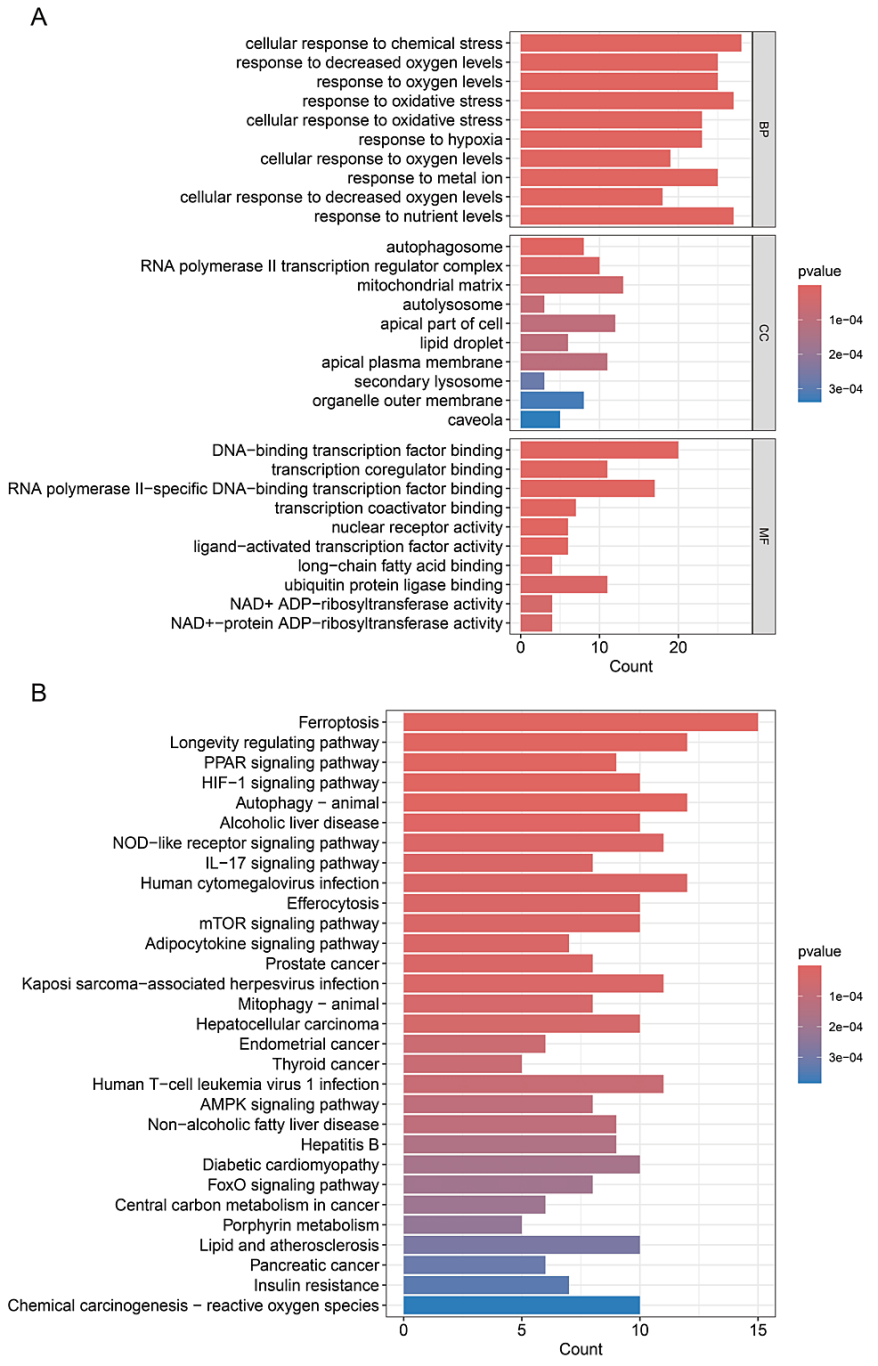
Enrichment analyses of differentially expressed ferroptosis-related genes (DE-FRGs) in sepsis-induced cardiomyopathy (SICM). (A) Gene ontology (GO) analysis of DE-FRGs in SICM.  The *P*-values indicating significance are shown along the *y*-axis, highlighting the processes most altered in SICM due to ferroptosis-related gene expression changes. (B) Kyoto Encyclopedia of Genes and Genomes (KEGG) Pathway Enrichment for DE-FRGs.

To elucidate the biological functions and pathways influenced by DE-FRGs in the context of SICM, we conducted GO and KEGG pathway enrichment analyses. The results from the GO analysis highlighted significant involvement of these genes in processes like response to oxidative stress, NAD^+^-protein ADP-ribosyltransferase activity, and transcriptional regulation, as shown in the corresponding bar plot (Figure 2A). KEGG analysis linked these genes predominantly with pathways including lipid metabolism, ferroptosis, and insulin resistance (Figure 2B). These findings suggest a robust association of SICM with cellular stress responses and metabolic dysregulation.

Identification of key DE-FRGs in SICM

**Figure 3:**
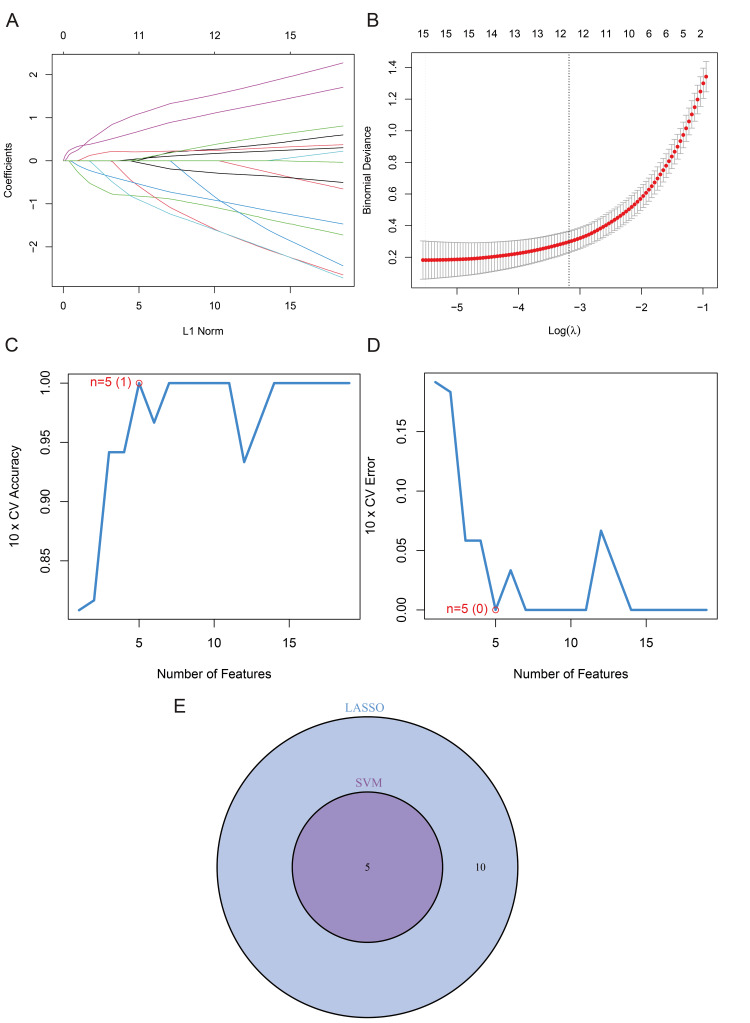
Identification of key differentially expressed ferroptosis-related genes (DE-FRGs) in sepsis-induced cardiomyopathy (SICM). (A) Optimization of the least absolute shrinkage and selection operator (LASSO) model's penalty parameter (λ) using 10-fold cross-validation. (B) Coefficient paths in the LASSO model. (C) and (D) The support vector machine with recursive feature elimination (SVM-RFE) algorithm identified a core set of five DE-FRGs based on the evaluation of cross-validation accuracy and error rate. (E) A Venn diagram depicts the intersection of marker genes derived from both the LASSO and SVM-RFE models.

By employing the least absolute shrinkage and selection operator (LASSO) and SVM-RFE algorithms, we identified key DE-FRGs associated with SICM. The LASSO method, optimized through 10-fold cross-validation, identified 15 DE-FRGs (Figures 3A-3B), while SVM-RFE pinpointed five genes that exhibited optimal discrimination (Figures 3C-3D). Integration of these results highlighted five key DE - FRGs-NCOA4, GABARAPL1, GJA1, CISD1, CP - as candidates for further analysis (Figure 3E).

Gene set enrichment analysis (GSEA) of key DE-FRGs

**Figure 4:**
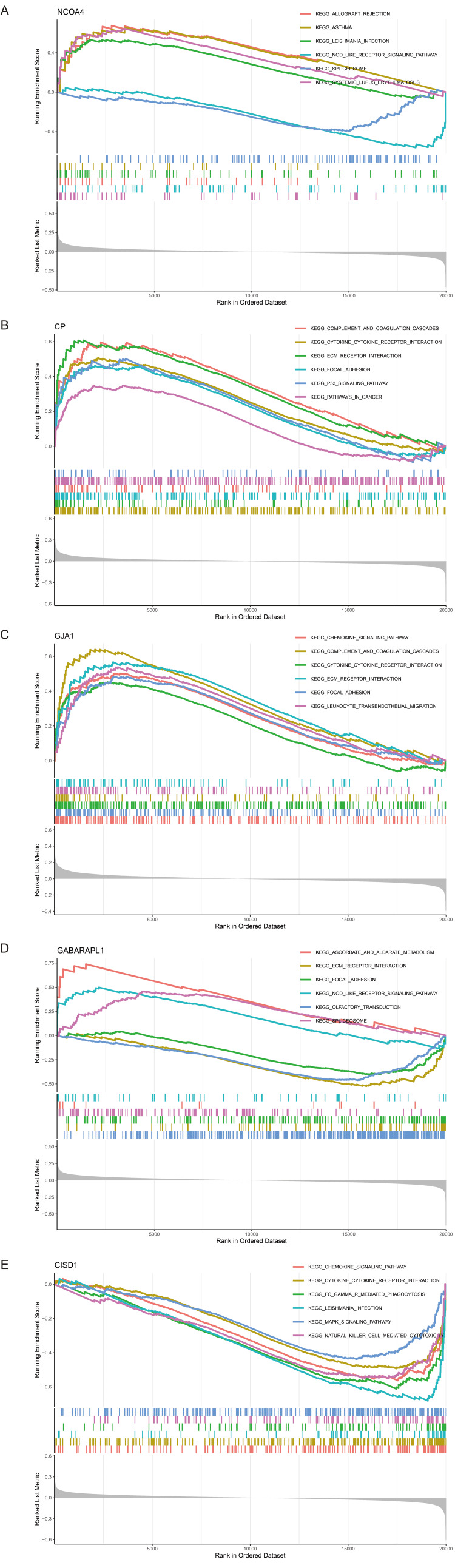
Gene set enrichment analysis (GSEA) of key differentially expressed ferroptosis-related genes (DE-FRGs) in sepsis-induced cardiomyopathy (SICM). (A) Nuclear receptor coactivator 4 (NCOA4) is shown to be involved in allograft rejection and nucleotide-binding oligomerization domain-like (NOD-like) receptor signaling pathways. (B) CP is associated with complement and coagulation cascades, focal adhesion, and cancer-related pathways. (C) GJA1 is associated with complement and coagulation cascades and focal adhesion. (D) GABARAPL1 is linked to autophagy, extracellular matrix (ECM) receptor interaction, and the spliceosome. (E) CISD1 is related to natural killer cell-mediated cytotoxicity and the mitogen-activated protein kinase (MAPK) signaling pathway.

To further elucidate the functional implications of the five key DE-FRGs, we performed GSEA targeting KEGG pathways. Notably, NCOA4 exhibited relevance with pathways concerning allograft rejection and NOD-like receptor signaling (Figure 4A), while CP was associated with complement and coagulation cascades, focal adhesion, and pathways in cancer (Figure 4B). Additionally, GJA1 was also significantly enriched in complement and coagulation cascades and focal adhesion (Figure 4C), and GABARAPL1 was linked to autophagy-related pathways, ECM receptor interaction, and spliceosome (Figure 4D). CISD1 was related to natural killer cell-mediated cytotoxicity and the MAPK signaling pathway (Figure 4E). These findings underscore the complex impact of these genes on SICM pathophysiology, linking them to a broad spectrum of biological processes from immune response to metabolic regulation.

Drug prediction for key DE-FRGs in SICM

**Figure 5:**
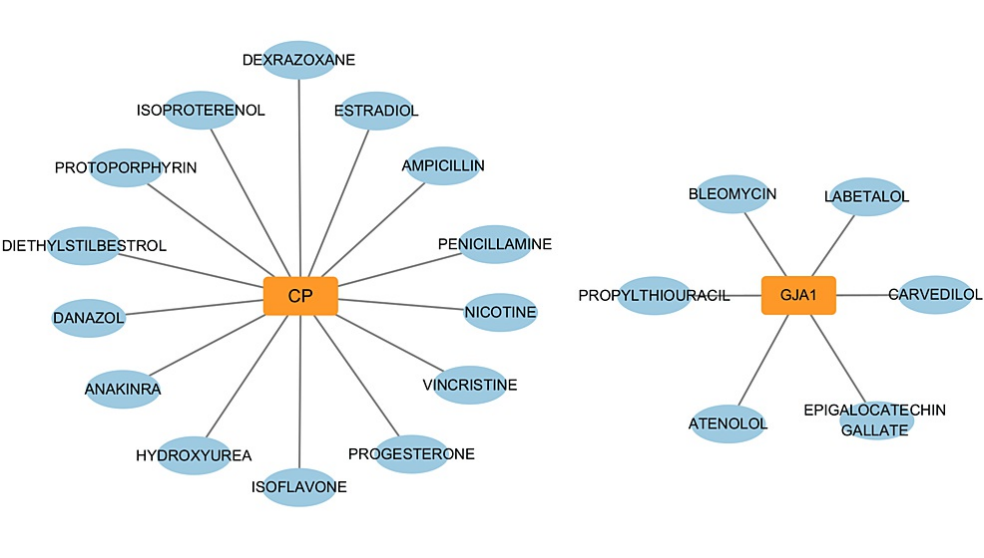
Predicted drug-gene interaction network for GJA1 and CP. This figure presents two distinct networks illustrating the potential therapeutic drugs that may interact with the genes GJA1 (top) and CP (bottom). For GJA1, the network includes drugs such as carvedilol, epigallocatechin gallate, and atenolol, indicating its potential involvement in pathways amenable to treatment with cardiovascular and antioxidant agents. In contrast, the network for CP shows a diverse set of drugs, including penicillamine, estradiol, and nicotine, reflecting CP's role in metabolic processes and stress responses that these drugs could modulate.

In our quest to identify potential therapeutic compounds targeting the DE-FRGs in SICM, we utilized the Drug Gene Interaction Database (DGIdb). This analysis led to the identification of 20 drugs that are predicted to interact with two of the hub DE-FRGs, specifically GJA1 and CP (Figure 5). For GJA1, potential interactions were identified with drugs such as carvedilol, epigallocatechin gallate, and atenolol, suggesting its involvement in pathways that may benefit from cardiovascular and antioxidant therapies. In contrast, CP was associated with a diverse array of drugs, including dexrazoxane, penicillamine, estradiol, and nicotine, which may modulate the metabolic processes and stress responses in which CP participates.

Construction of a ceRNA network centered on key DE-FRGs

**Figure 6:**
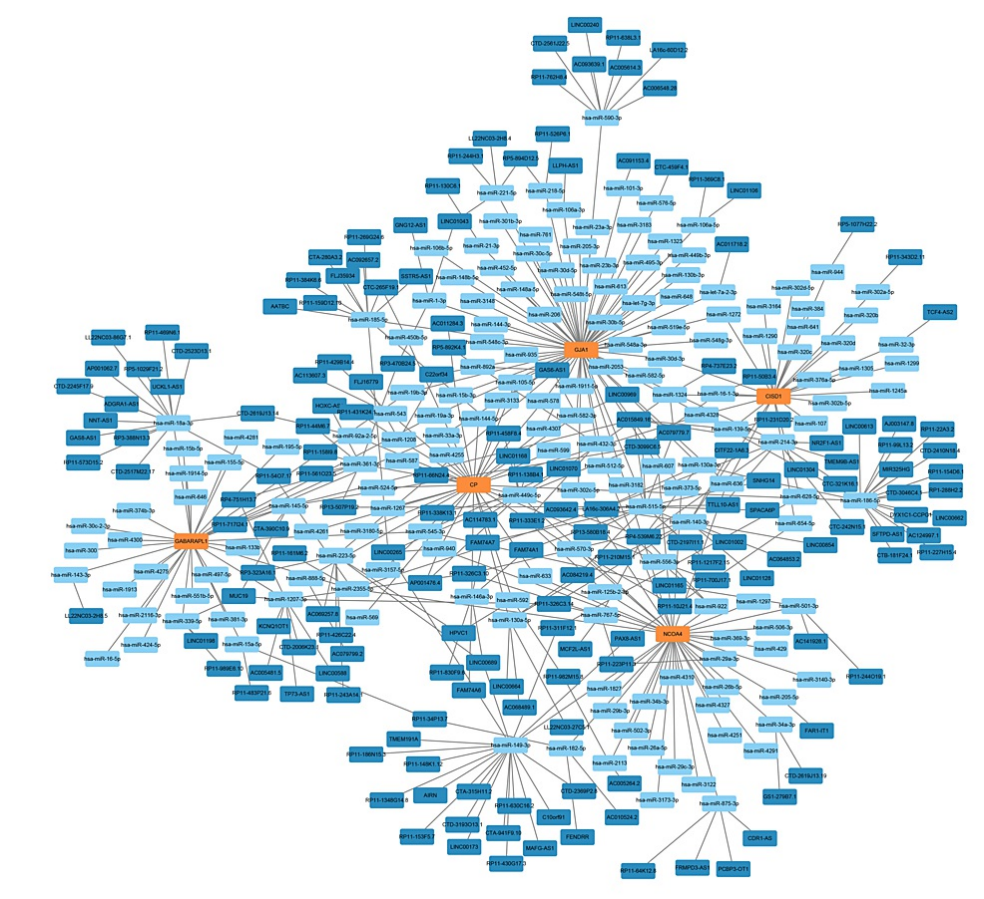
ceRNA network highlighting the regulatory interplay among differentially expressed ferroptosis-related genes (DE-FRGs), microRNAs (miRNAs), and long non-coding RNAs (lncRNAs) This network illustrates the intricate web of interactions where lncRNAs may modulate the expression of key DE-FRGs, including through competitive binding with miRNAs.

In our further exploration, a ceRNA network was constructed to understand the regulatory landscape surrounding key DE-FRGs. This network encompassed 439 nodes, which included the central key DE-FRGs, as well as 196 miRNAs and 238 lncRNAs. This intricate network map, as visualized in Figure 6, demonstrated the potential regulatory triads where, for example, GABARAPL1 expression was putatively controlled by the lncRNA MUC19 through its competitive interactions with specific miRNAs such as hsa-miR-339-5p, hsa-miR-1207-3p, and hsa-miR-145-5p. The network also suggests potential regulation of NCOA4 expression by lncRNAs competing with miRNAs like hsa-miR-501-3p, hsa-miR-205-5p, and hsa-miR-34a-3p. A prominent lncRNA, RP4-539M6.22, emerged as a potential regulatory hub, capable of influencing the expression of multiple DE-FRGs, including NCOA4 and GJA1, via interactions with a distinct set of miRNAs. This ceRNA network offers a valuable framework for further investigation into the post-transcriptional regulatory mechanisms involved in ferroptosis and their contribution to the pathogenesis of SICM.

## Discussion

This study has delved into the complex pathophysiological mechanisms underpinning SICM, with a particular focus on the role of ferroptosis - a recently characterized form of programmed cell death driven by iron-dependent lipid peroxidation [5]. While substantial progress has been made in understanding SICM, the molecular mechanisms and prognostic markers that could guide diagnostics and therapeutic strategies remain elusive. Ferroptosis has been implicated in a variety of cardiovascular diseases and is now recognized as a critical factor contributing to the progression of SICM [12,13].

Our systematic bioinformatics approach led to the identification of five key DE-FRGs that may serve as predictive biomarkers for SICM (NCOA4, GABARAPL1, GJA1, CISD1, CP), aligning with their established roles in regulating ferroptosis. In a recent study, NCOA4 was highlighted as a key regulator in iron metabolism during SICM, specifically through its role in the autophagic degradation of ferritin [12]. This process is crucial for maintaining iron homeostasis within cells, and its dysregulation can lead to increased free iron levels [12]. Moreover, the other identified genes - GABARAPL1, GJA1, CISD1, and CP - are implicated in diverse biological processes, such as autophagy, signaling, mitochondrial function, and iron metabolism, suggesting their composite involvement in SICM development [14-16].

The identification of potential therapeutic compounds, such as dexrazoxane, an inhibitor known to mitigate ferroptosis [17], provides new avenues for future research. Dexrazoxane has demonstrated efficacy in reducing mitochondrial damage and enhancing cardiac function in sepsis models, underscoring its potential utility in ameliorating sepsis-induced cardiac injuries [12]. Additionally, the construction of a comprehensive ceRNA network provided insights into the post-transcriptional regulatory mechanisms for DE-FRGs during SICM, emphasizing the critical interplay between coding and non-coding RNAs in gene expression regulation. These findings offer a promising avenue for the development of novel treatment strategies for SICM.

Despite the insights gained from this study, some limitations must be acknowledged. The retrospective nature of the analyzed data and the focus on a specific subset of genes may have introduced bias. Additionally, the applicability of our findings across different ethnic groups remains to be validated, given that the study participants of the GSE79962 dataset may not fully represent the global population diversity seen in SICM cases. Therefore, further empirical studies, including in vitro and in vivo experiments, are essential to thoroughly ascertain the roles of those DE-FRGs in SICM pathogenesis.

## Conclusions

This investigation enhances our understanding of the molecular underpinnings of SICM by pinpointing key DE-FRGs and elucidating their roles in disease progression. These findings lay a solid foundation for future research aimed at developing targeted therapeutic and diagnostic approaches for SICM. As we continue to dissect the complex relationship between ferroptosis and sepsis-induced cardiac dysfunction, our study paves the way toward a more comprehensive understanding and effective management of SICM.
